# Cytoreductive surgery for synchronous and metachronous colorectal peritoneal dissemination: Japanese P classification and peritoneal cancer index

**DOI:** 10.1002/ags3.12721

**Published:** 2023-07-19

**Authors:** Akiyoshi Mizumoto, Nobuyuki Takao, Toru Imagami, Byonggu An, Yasumitsu Oe, Takeshi Togawa, Yutaka Yonemura

**Affiliations:** ^1^ Department of Gastrointestinal Surgery and Peritoneal Dissemination Center, Omi Medical Center Kusatsu Japan; ^2^ NPO to support Peritoneal Surface Malignancy Treatment Kyoto Japan

**Keywords:** classification, colorectal cancer, cytoreductive surgery, PCI, peritoneal carcinomatosis

## Abstract

**Aim:**

The outcomes of cytoreductive surgery (CRS) for synchronous and metachronous colorectal peritoneal dissemination were investigated using the Japanese P classification and peritoneal cancer index (PCI).

**Methods:**

CRS was performed in 111 cases of synchronous peritoneal dissemination and 115 cases of metachronous peritoneal dissemination. The P classification and PCI were determined at the time of laparotomy.

**Results:**

In the synchronous dissemination group, the 5‐year overall survival rates after CRS in P1/P2 and P3 cases were 51% and 13%, respectively. Even for P3, 51% of the patients achieved macroscopic cytoreductive complete resection (CC‐0), with a 5‐year survival rate of 40%. When P3 cases were classified into PCI 0–9, 10–19, 20–29, and 30–39, CC‐0 was achieved in 93%, 70%, 6%, and 0% of the cases, respectively, and the 5‐year survival rate of PCI 0–9 was 41%. In the metachronous dissemination group, the 5‐year survival rates were 62% for PCI 0–9 and 22% for PCI 10–19; 5‐year survival was not observed in patients with a PCI ≥ 20. CC‐0 was significantly associated with the postoperative prognosis in both synchronous and metachronous peritoneal dissemination.

**Conclusion:**

In cases of synchronous dissemination, CRS must be performed for P1 and P2 cases or those with a PCI < 10, while detailed examination using PCI is required for P3 cases. In cases of metachronous dissemination, CRS should be considered when the PCI score is <20.

## Graphical abstract

Overall survival curves after CRS for synchronous and metachronous peritoneal dissemination group.

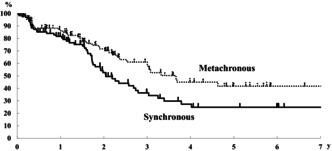

## 1 INTRODUCTION

Colorectal peritoneal dissemination has a poor prognosis. The incidence of synchronous and metachronous peritoneal dissemination after R0 resection is 4%–8% and approximately 4%, respectively.^1^ Despite recent advances in chemotherapy, patients with peritoneal dissemination do not show a significant improvement in prognosis compared to those with distant metastases to the liver, lungs, and lymph nodes.^2^

In synchronous colorectal peritoneal dissemination, the P classification is widely used in Japan because it is simple and reflects the prognosis well.^3^ Complete resection is strongly recommended for P1 (metastasis localized to the adjacent peritoneum), while complete resection, if resectable, is recommended for P2 (limited metastasis to the distant peritoneum). Meanwhile, the efficacy of resection for P3 (diffuse metastasis to the distant peritoneum) has not been established according to the Japanese Guidelines for the Treatment of Colorectal Cancer.^4^

The peritoneal cancer index (PCI) is a quantitative method that is widely used^5^ and is associated with the possibility of complete cytoreduction and postoperative prognosis.

For metachronous colorectal peritoneal dissemination, surgical resection is considered if complete resection is possible. However, there are only few studies on the possibility of surgical resection based on the P classification and PCI.

Recently, reports have shown that cytoreductive surgery (CRS) is useful for peritoneal dissemination of colorectal cancer. Some international guidelines consider CRS a treatment option for colorectal dissemination when complete resection is possible.

In this study, we showed the outcomes of CRS for synchronous and metachronous dissemination of colorectal cancer and examined the indications for CRS according to the P classification and PCI.

## 2 MATERIALS AND METHODS

Patients with uncontrollable distant metastasis other than peritoneal dissemination, extensive dissemination in the small intestine, and a performance status worse than 3 were excluded from this study. Overall, 226 patients who underwent CRS for peritoneal dissemination of colorectal cancer between November 2012 and August 2021 were included in the study. There were 111 and 115 cases of synchronous and metachronous peritoneal dissemination, respectively.

Regarding the Japanese P classification, P1, P2, and P3 were determined based on findings during laparotomy for both synchronous and metachronous dissemination at our hospital. P1 referred to disseminated metastases confined to the proximal peritoneum of the primary tumor, P2 indicated a few disseminated metastases in the distant peritoneum, and P3 denoted the presence of multiple disseminated metastases in the distant peritoneum.^3^

PCI was determined at the time of laparotomy.^5^ The abdominal cavity was divided into nine sections, while the small intestine was divided into four sections. A score of 0 was given if no tumor was present, 1 if the tumor measured <5 mm in the greatest dimension, 2 if it measured between 5 mm and 5 cm, and 3 if it measured >5 cm. For further analysis, the PCI was divided into four groups (PCI 0–9, PCI 10–19, PCI 20–29, and PCI 30–39), and the postoperative outcomes were examined in each group.

CRS was performed by combining greater and lesser omentectomies, parietal peritoneal resection, and organ resection based on Sugarbaker's method.^6^ CRS was performed for the first time at our institution, regardless of whether the standard resection surgery for disseminated lesions was performed at the previous facility. The degree of resection was expressed as the completeness of the cytoreduction (CC) score. CC‐0 is grossly free of a residual tumor, CC‐1 is a residual tumor <2.5 mm, CC‐2 is residual tumor measuring 2.5 mm–2.5 cm, and CC‐3 is a residual tumor >2.5 cm.^5^

Preoperative chemotherapy consisted of various regimens, including XELOX (capecitabin, oxaliplatin); SOX (S‐1, oxaliplatin); FOLFOX; FOLFIRI; IRIS (irinotecan, S‐1); and oral chemotherapy agents. Patients received a minimum of three cycles of chemotherapy. Adjuvant chemotherapy was initiated 4–8 weeks after CRS, and regimens consistent with the aforementioned chemotherapy options were administered. Postoperative chemotherapy was typically administered for 6–12 months or more, but cases that received a minimum of three cycles of chemotherapy were also included in the postoperative chemotherapy group.

Hyperthermic intraperitoneal chemotherapy (HIPEC) was administered following CRS. The intraperitoneal cavity was washed with 10 L (1 L × 10 times) of normal saline. Two infusion tubes were placed in the upper abdomen, and one drainage tube was placed in the pelvic cavity. HIPEC was perfused for 1 h with 42°C–43°C normal saline containing 20 mg of mitomycin. After completion of HIPEC, the peritoneal cavity was washed again with 10 L (1 L × 10 times) of normal saline. HIPEC was performed on 141 patients only until 2019 owing to issues with the Japanese insurance system. This treatment method was approved by the Ethics Review Committee of our hospital (2012–1026‐1).

The presence or absence of HIPEC may have influenced the prognosis and related factors. Consequently, we examined the difference in survival rates between the synchronous and metachronous peritoneal dissemination groups, both with and without HIPEC. The Kaplan–Meier survival curve was used to analyze the survival rates. The generalized Wilcoxon test was used to determine significance, and the Cox proportional hazards test was used for multivariate analysis. Statistical significance was set at *p* < 0.05.

## 3 RESULTS

### 3.1 Comparison of synchronous and metachronous dissemination group

The details of the cases are listed in Table 1. There were no significant differences in sex, age, or primary site between synchronous and metachronous cases. In the synchronous peritoneal dissemination group, there were significantly more cases of primary appendiceal cancer and more undifferentiated types such as poorly differentiated, signet ring cells, and mucinous.

**TABLE 1:** Comparison between synchronous and metachronous dissemination group.

	Synchronous group (*n* = 111)	Metachronous group (*n* = 115)	Synchronous vs. metachronous
Male/Female	52/59	51/64	NS
Age	53 ± 14 (21–83)	56 ± 13 (30–78)	NS
Primary site; right/left‐sided	32/43	44/60	NS
Appendix/Colorectal	36/75	11/104	*p* < 0.001
Histology; differentiated/undifferentiated	42/69	79/36	*p* < 0.001
pT2/pT3/pT4/unknown	0/20/78/13	1/43/64/7	NS
Deeper than pT3	100%	99%	
pN0/pN1/pN2/pN3/unknown	21/31//31/7/21	39/42/2 0/ 5/9	NS
Positive LN metastasis	77%	62%	
Preoperative CEA (mean ± SE)	60 ± 179 ng/mL	50 ± 176 ng/mL	NS
Preoperative CA19‐9 (mean ± SE)	289 ± 1367 U/mL	167 ± 729 U/mL	NS
From diagnosis to CRS (month)	12 ± 11 (0–58)	12 ± 9 (0.4–58)	NS
Within 6 months to CRS; *n* (%)	48 (43%)	51 (44%)	NS
P classification; P1/P2/P3	5/21/85	5/37/73	NS
Peritoneal cancer index (PCI)	15 ± 11	12 ± 9	*p* < 0.05
CC‐0/CC‐1,2,3	57/54	86/29	*p* < 0.05
Operation time (mean ± SE)	286 ± 93 min	290 ± 86 min	NS
Bleeding volume (mean ± SE)	1.2 ± 0.8 L	1.3 ± 0.9 L	NS
Postoperative complication; Grade 3≧	24 (22%)	26 (23%)	NS
Preoperative chemotherapy; yes/no/unknown	83/27/1	103/11/1	*p* < 0.05
Adjuvant chemotherapy; yes/no/unknown	77/11/23	76/12/27	NS
HIPEC; done/not done	65/46	76/39	NS

In almost all cases (both the synchronous and metachronous dissemination groups), the T factor of the primary tumor exceeded T3. Lymph node metastases were present in 77% and 62% of patients in the synchronous and metachronous groups, respectively; however, there were some cases with unknown lymph node status. The median interval between a definite diagnosis of peritoneal dissemination and CRS was 12 months in both groups, and CRS was performed within 6 months in 43% and 44% of the patients in the synchronous and metachronous dissemination groups, respectively.

The mean intraoperative PCI was 15 ± 11 in the synchronous group and 12 ± 9 in the metachronous group; the difference between groups was significant. No significant differences were observed in the operative time and bleeding volume between the groups.

Grade 3 or higher postoperative complications occurred in 24 (22%) and 26 (23%) patients in the synchronous and metachronous dissemination groups, respectively. The most common complication in the synchronous dissemination group was intraabdominal abscess (10 patients), followed by postoperative bleeding, anastomotic leakage, and intestinal perforation (three patients each). The most common complication in the metachronous dissemination group was also intraabdominal abscess (12 patients), followed by postoperative bleeding (four patients), anastomotic leakage (three patients), and urinary bladder fistula (three patients each).

Preoperative chemotherapy before CRS was performed in 75% and 90% of the patients in the synchronous and metachronous groups, respectively. Postoperative chemotherapy after CRS was performed in most cases in both groups, although there were some cases of unknown administration status. HIPEC was performed in 65 of 111 and 76 of 115 patients in the synchronous and metachronous dissemination groups, respectively.

**FIGURE 1:**
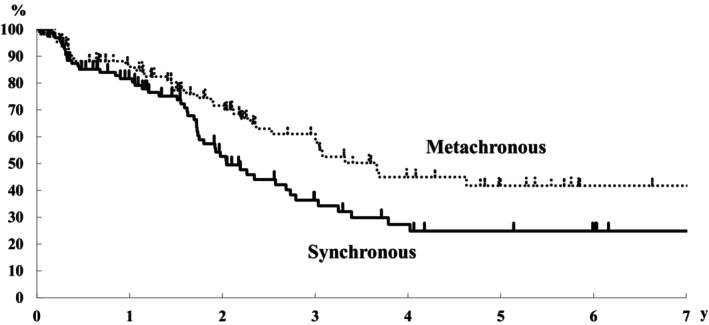
Overall survival curves of the synchronous and metachronous peritoneal dissemination groups.

The MSTs of the synchronous and metachronous dissemination group were 25 and 44 months, and the 3‐ and 5‐year survival rates were 36% and 61% and 25% and 42%, respectively (Figure 1). The survival rate tended to be more favorable in the metachronous dissemination group; however, no significant differences were observed.

### 3.2 Synchronous dissemination group

**FIGURE 2:**
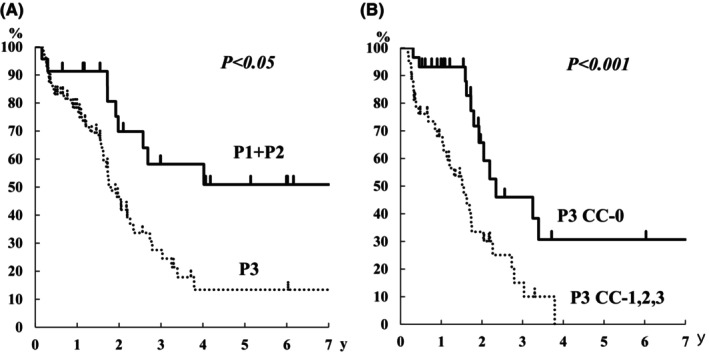
Overall survival curves of P1 + P2 and P3 cases in the synchronous peritoneal dissemination group. Overall survival curves of P3 cases with CC‐0 and CC‐1, ‐2, and ‐3 in the synchronous peritoneal dissemination group.

There were five cases of P1, 21 of P2, and 85 of P3 in the synchronous dissemination group (Table 1). CC‐0 was achieved in 100% (5/5) of cases of P1, 95% (20/21) of P2, and 38% (32/85) of P3. The 3‐ and 5‐year overall survival rates for P1 + P2 cases were 58% and 51%, respectively (Figure 2A). Patients with P3 had an MST of 22 months, and their 3‐ and 5‐year overall survival rates were 28% and 13%, respectively (Figure 2A). When CC‐0 was achieved in P3 cases, the MST was 28 months, and the 3‐ and 5‐year overall survival rates were 46% and 31%, respectively (Figure 2B).

**FIGURE 3:**
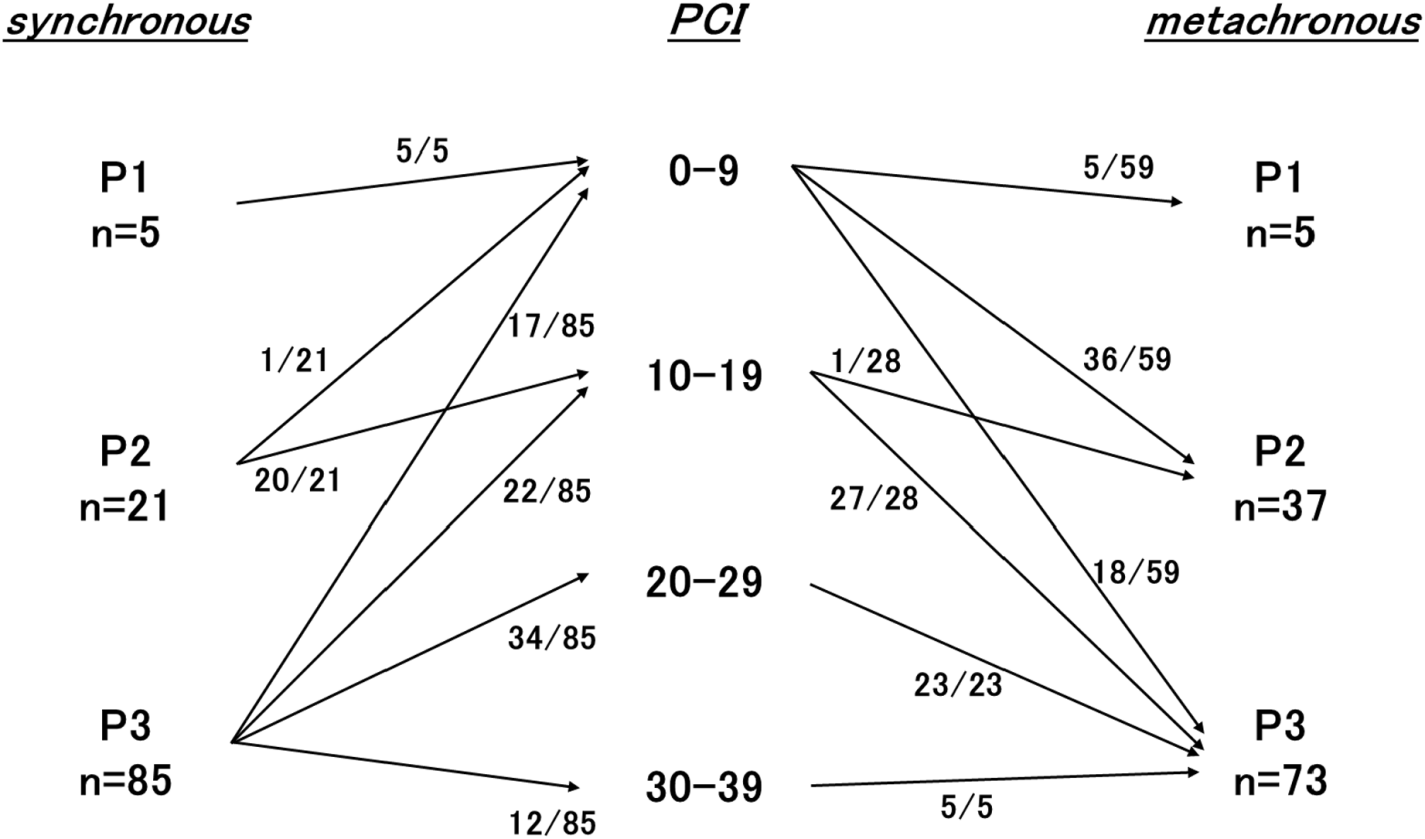
Correspondence between the P classification and PCI in the synchronous and metachronous dissemination groups.

**FIGURE 4:**
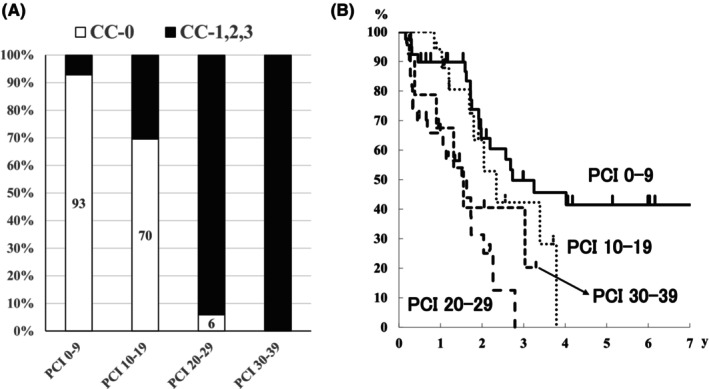
Percentage of CC‐0 and CC‐1, ‐2, and ‐3 in each PCI group in patients with synchronous peritoneal dissemination. Overall survival curves of each PCI group in patients with synchronous peritoneal dissemination.

According to the correspondence between the P classification and PCI (Figure 3), P1 and P2 cases were classified as PCI 0–9 and PCI 10–19, respectively, except for one case of P2. Meanwhile, P3 cases were included in all four PCI groups. CC‐0 was achieved in 93% of the cases with PCI 0–9 and 70% of the cases with PCI 10–19 (Figure 4A). However, CC‐0 was only achieved in 6% of the cases with PCI 20–29, and no cases with PCI 30–39 achieved CC‐0 (Figure 4A). Considering the survival rates on basis of the PCI groups, PCI 0–9 group comprised long‐term survivors (Figure 4B).

### 3.3 Metachronous dissemination group

**FIGURE 5:**
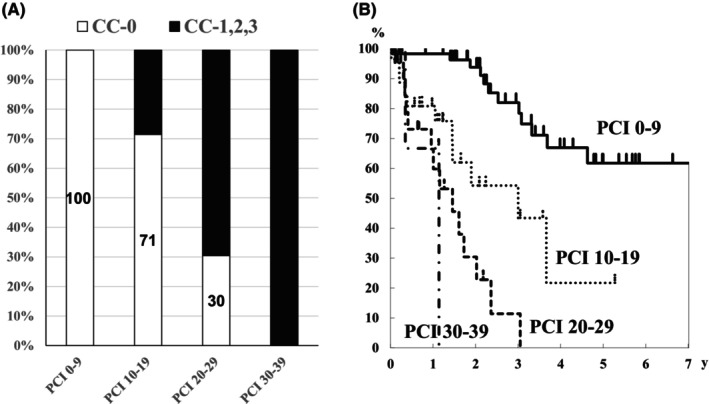
Percentage of CC‐0 and CC‐1, ‐2, ‐3 in each PCI group in patients with metachronous peritoneal dissemination. Overall survival curves of each PCI group in patients with metachronous peritoneal dissemination.

In the metachronous dissemination group, CC‐0 was achieved in all patients with PCI 0–9, 71% with PCI 10–19, and 30% with PCI 20–29; CC‐0 was not achieved in cases wherein the PCI was ≥30 (Figure 5A). Considering the survival rate, long‐term survival was observed in patients with a PCI < 20 (Figure 5B).

**FIGURE 6:**
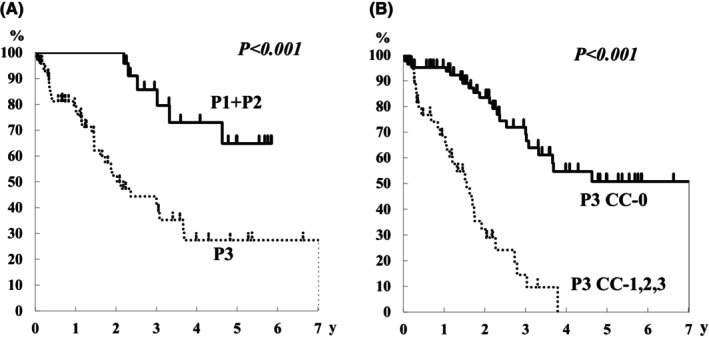
Overall survival curves of P1 + P2 and P3 cases in the metachronous peritoneal dissemination group. Overall survival curves of P3 cases with CC‐0 and CC‐1, ‐2, and ‐3 in the metachronous peritoneal dissemination group.

When each PCI group was categorized based on the P classification, PCI 0–9 was classified as P1–P3, and all cases exceeding PCI 10 were classified as P3, except for one case (Figure 3). Considering the survival rate according to the P classification for metachronous disease, the 5‐year overall survival rate for P1/P2 and P3 cases were 65% and 27%, respectively (Figure 6A). When CC‐0 was achieved in P3 cases, the 3‐ and 5‐year overall survival rates were 72% and 51%, respectively (Figure 6B).

### 3.4 Factors affecting prognosis in the synchronous and metachronous dissemination groups (Table 2)

**TABLE 2:** Univariate and multivariate analysis for synchronous and metachronous dissemination group.

	Synchronous group			Metachronous group		
	Univariate analysis	Multivariate analysis		Univariate analysis	Multivariate analysis	
	*p*‐Value	HR (95% CI)	*p*‐Value	*p*‐Value	HR (95% CI)	*p*‐Value
Male vs. Female	0.389			0.913		
Age; <65 vs. ≥65	0.407			0.875		
Primary site; right‐sided vs. left‐sided	0.993			0.743		
Appendix vs. Colorectal	0.383			0.116		
Histology; differentiated vs. undifferentiated	0.451			0.418		
LN metastasis; negative vs. positive^a^	0.838^a^			0.518^a^		
Preoperative CEA; <6.0 vs. ≥20 ng/mL	0.026	0.949 (0.468–1.923)	0.884	0.059		
Preoperative CA19‐9; <37 vs. ≥37 U/mL	0.012	0.718 (0.380–1.356)	0.307	<0.001	0.363 (0.185–0.712)	0.003
From diagnosis to CRS; <6 months vs. ≥6 months	0.018	0.493 (0.260–0.934)	0.030	0.545		
Peritoneal cancer index (PCI); <20 vs. ≥20	<0.001	0.856 (0.386–1.897)	0.701	<0.001	0.264 (0.112–0.622)	0.002
CC‐0 vs. CC‐1,2,3	<0.001	0.320 (0.141–0.728)	0.007	<0.001	0.401 (0.180–0.890)	0.025
Postoperative complications	0.120			0.073		
HIPEC; done vs. not done	0.840			0.248		
Preoperative chemotherapy; yes vs. no	0.331			0.810		
Adjuvant chemotherapy^a^; yes vs. no	0.001^a^			<0.001^a^		

^a^ Reference values excluding unknown cases.

In the synchronous peritoneal dissemination group, univariate analysis showed that preoperative CEA and CA19‐9 levels within the normal range, PCI < 20, time to CRS <6 months, and CC‐0 were significantly favorable prognostic factors. Preoperative chemotherapy had no effect on the postoperative prognosis. In contrast, patients who received adjuvant chemotherapy had a significantly better prognosis than those who did not. However, the statistical result should be interpreted with caution owing to uncertainty regarding the administration of adjuvant chemotherapy in some patients. Additionally, postoperative complications did not affect the postoperative prognosis. In the multivariate analysis, time to CRS of <6 months and CC‐0 were significantly associated with a favorable prognosis.

In the metachronous dissemination group, preoperative CA19‐9 levels within the normal range, PCI < 20, and CC‐0 were significant prognostic factors on both univariate and multivariate analyses. Meanwhile, postoperative complications tended to worsen the postoperative prognosis (*p* = 0.073). Similar to the synchronous group, patients who received adjuvant chemotherapy had a significantly better prognosis than those who did not. However, the statistical findings should be considered indicative owing to uncertainty regarding the administration of adjuvant chemotherapy in certain patients.

### 3.5 Comparison of prognosis between CRS with HIPEC and CRS alone

In both synchronous and metachronous peritoneal dissemination groups, there was no significant difference in prognosis based on the presence or absence of HIPEC (Table 2).

Among the patients in the synchronous peritoneal dissemination group who underwent CRS alone, preoperative CEA (*p* = 0.019) and CA19‐9 (*p* = 0.032) within the normal range, time to CRS <6 months (*p* = 0.009), PCI < 20 (*p* = 0.022), and CC‐0 (*p* = 0.009) were significantly favorable prognostic factors. Among the patients who underwent CRS with HIPEC in the synchronous peritoneal dissemination group, PCI < 20 (*p* = 0.006) and CC‐0 (*p* = 0.001) were the only significantly favorable prognostic factors, whereas preoperative CEA (*p* = 0.247) and CA19‐9 (*p* = 0.062) within the normal range and time to CRS <6 months (*p* = 0.240) were not considered significant.

Among the patients who underwent CRS alone in the metachronous peritoneal dissemination group, preoperative CA19‐9 (*p* = 0.001) within the normal range, PCI < 20 (*p* = 0.047), and CC‐0 (*p* = 0.005) were significantly favorable prognostic factors. Among the patients who underwent CRS with HIPEC in the metachronous peritoneal dissemination group, preoperative CA19‐9 (*p* = 0.004) within the normal range, PCI < 20 (*p* < 0.001), and CC‐0 (*p* < 0.001) were also significantly favorable prognostic factors.

## 4 DISCUSSION

Peritoneal dissemination of colorectal cancer has a poor prognosis, with an MST of approximately 6 months without treatment. Although current advances in chemotherapy have improved prognosis, it is not as effective in cases of distant metastasis to other organs (liver, lungs, and lymph nodes).^2^ Over the last 30 years, treatment with CRS with HIPEC has reportedly improved the prognosis of disseminated colorectal cancer. In a randomized trial by Verwaal et al.,^7^ the MST following CRS with HIPEC was 22.3 months but that following systemic chemotherapy and palliative surgery was 12.6 months. In a multi‐institutional study, Glehen et al.^8^ reported a favorable MST of 32.4 months when complete resection was performed. A meta‐analysis also reported that the 5‐year overall survival rate following CRS with HIPEC was 19%–51% compared to 5%–13% for chemotherapy alone.^9^ In the synchronous and metachronous dissemination groups in this study, the MST after CRS was 25 and 44 months, while the 5‐year overall survival rates were 25% and 42%, respectively. The relatively better survival with metachronous dissemination compared to synchronous dissemination may be related to the significantly more histologically differentiated types, significantly lower PCI, and significantly higher number of cases that achieved CC‐0. Among cases of metachronous peritoneal dissemination with PCI 20–29, the proportion that achieved CC‐0 was higher compared to that among cases of synchronous peritoneal dissemination with PCI 20–29 (30% vs. 6%). In this study, 26 of 34 and 11 of 23 patients with PCI 20–29 in synchronous and metachronous dissemination, respectively, had undifferentiated histology. The exact reasons remain unclear; however, there is a possibility that the higher prevalence of undifferentiated cases of synchronous peritoneal dissemination may have been the contributing factor. This could be attributed to the possibility of more widespread and diffuse disseminated lesions, a higher occurrence of small bowel involvement, or a stronger tendency for invasion into organs in synchronous dissemination.

### 4.1 Synchronous peritoneal dissemination

The Japanese P classification is a simple method that accurately reflects prognosis. A previous study showed 5‐year survival rates of 26% for P1, 12% for P2, and 7% for P3.^10^ However, recent advances in surgical techniques and the accumulation of knowledge have changed our way of thinking about surgical approaches to peritoneal dissemination. In fact, Japanese Colorectal Cancer Treatment Guidelines strongly recommend complete resection of P1 and P2 when easily resectable.^4^ In this report, CC‐0 was achieved in almost all P1/P2 cases, with a 5‐year survival rate of 51%. The difference in survival rates between the previous report^10^ and our study may be owing to difference in the surgical techniques between conventional surgery used in the previous study and CRS performed in our study. Namely, the favorable survival rate in this study may be because peritonectomy can achieve a higher CC‐0 rate than ordinary surgical resection. CRS can be performed to remove diffuse and extensive peritoneal metastases. In fact, CC‐0 was achieved in 96% of the P1/P2 cases in this study, whereas a previous study showed that R0/R1 was obtained in 53% of P1 and 44% of P2 cases.^11^

P3 is rarely considered as an indication for resection in Japan. However, this study showed that CC‐0 could be achieved even in P3 cases; when CC‐0 was achieved, the 5‐year overall survival rate was 31%. Therefore, the selection of P3 cases for CRS is critical.

Regarding the classification of PCI into four groups and examining their relationship with the P classification, P1 and P2 cases all had a PCI < 10, except for one case of P2. Conversely, P3 cases were included in all four PCI groups. CC‐0 was achieved in 93% of the cases with PCI 0–9 and in 70% with PCI 10–19. However, when the PCI was >20, CC‐0 was not achieved in most cases. Therefore, in patients with synchronous peritoneal dissemination, CRS should be strongly considered at PCI 0–9 and PCI 10–19, carefully considered at PCI 20–29, and not considered at PCI ≥ 30.

Kawasaki et al.^12^ proposed a new P classification as follows: P1 is metastases confined to one peritoneal region, P2 is ≤19 peritoneal metastases in two or more regions, and P3 is ≥20 metastases in two or more regions. The authors suggest that the association between this new P classification system and prognosis should be investigated in the future. We believe that the use of the PCI is desirable because it can compare results with those in other countries and that the PCI should at least be used to determine indications for resection when the patient is confirmed to have P3.

### 4.2 Metachronous peritoneal dissemination

CC‐0 was achieved in 100% of the cases with PCI 0–9 and in 71% with PCI 10–19. Additionally, the 3‐ and 5‐year survival rates of patients with PCI 0–9 and PCI 10–19 were 82% and 62% and 54% and 22%, respectively. In contrast, CC‐0 was achieved in 30% of patients with PCI 20–29, and the 3‐year survival rate was 11%; no 5‐year survival was observed. Notably, if the PCI was ≥30, CC‐0 was not achieved, and 2‐year survival was not observed.

Although the P classification is not usually used for metachronous dissemination, each case was assigned to the P classification, and survival rates were examined. The 3‐ and 5‐year survival rates were 86% and 65% for P1/P2 cases, and 44% and 27% for P3 cases, respectively. The P classification is useful for predicting prognosis even in metachronous dissemination. Comparing the PCI and P classification, PCI = 0–9 was classified into P1–P3, and almost all cases with a PCI > 10 were classified as P3.

In cases of metachronous dissemination, guidelines do not indicate what cases may undergo resection. We showed that CRS can be considered an absolute indication if the P1 and P2 or the PCI is <20. If the PCI is 20–30, it is necessary to carefully determine the indications for CRS, and if the PCI is ≥30, CRS is not indicated.

### 4.3 Factors affecting survival after CRS

In synchronous dissemination, significant prognostic improvement was observed when the preoperative CEA or CA19‐9 values were within the normal range, PCI was <20, CC‐0 was achieved, or time to CRS was <6 months. Meanwhile, for metachronous dissemination, pre‐CRS CA19‐9 levels within the normal range, PCI < 20, and achievement of CC‐0 were significant prognostic factors.

Reports have shown that the CC score and PCI are the most important prognostic factors after CRS for colorectal peritoneal dissemination. Sugarbaker et al.^13^ showed that the MST in patients with PCI < 20 was significantly longer than those with PCI > 20. Elias et al.^14^ reported that PCI > 15 was the threshold for a significantly poor prognosis. Yonemura et al.^15^ also reported that PCI ≤ 10 was an independent prognostic factor.

Preoperative CA19‐9 levels are significantly associated with the prognosis of colorectal cancer,^16^ and pre‐CRS CA19‐9 levels may be similarly associated with post‐CRS prognosis for synchronous and metachronous peritoneal dissemination. Yang et al.^17^ showed that CA19‐9 and CA125 were independent variables for overall survival in a multivariate analysis. In contrast, Huo et al.^18^ showed that serum CEA and CA125 levels in patients with colorectal peritoneal dissemination treated with CRS with HIPEC had a negative prognostic effect independent of the PCI. Further investigations are needed regarding tumor markers and prognosis after CRS.

CRS with HIPEC is highly invasive and is associated with high morbidity and mortality rates. In this report, the incidence of postoperative complications of Grade 3 or higher exceeded 20%, and there were two cases (0.1%) of mortality within 30 days after CRS. A systematic review of the literature revealed morbidity and mortality rates of 22%–76% and 0%–19%, respectively.^19^ A recent report compared the morbidity and mortality rates following CRS with HIPEC with other high‐risk surgical oncological procedures.^20^ They showed that CRS with HIPEC was associated with a similar or lower morbidity rate than pancreatoduodenectomy, right lobe hepatectomy, or esophagectomy. Additionally, the 30‐day mortality rate was lower in CRS with HIPEC (1.1%) compared with Whipple (2.5%), right lobe hepatectomy (2.9%), and esophagectomy (3.0%).^20^

It has been reported that postoperative complications after CRS with HIPEC are associated with a poor oncological prognosis,^21^ and grades 3–4 postoperative complications correlated with worse overall survival.^22^ Postoperative complications may worsen the general condition and cause delays in the initiation of postoperative chemotherapy. In this study, postoperative complications did not affect the prognosis of patients in the synchronous dissemination group, while it tended to worsen the prognosis of patients in the metachronous dissemination group. The lack of significant difference in this study may be attributed to the limited number of cases included.

Interestingly, the prognosis was significantly better if the time to CRS was <6 months in the synchronous peritoneal dissemination group. Among the 48 patients who underwent CRS within 6 months in the synchronous dissemination group, 24 (50%) received preoperative chemotherapy. Meanwhile, among the 63 patients who underwent CRS after 6 months following the detection of peritoneal metastasis, chemotherapy was administered in 58 cases (92%). Theoretically, preoperative chemotherapy would be more likely to achieve tumor size reduction, reduce the extent of resection, and increase the rate of achieving CC‐0 if chemotherapy is effective. Some patients who received CRS after 6 months may have a poor response to chemotherapy, and an earlier consideration of performing CRS following the diagnosis of dissemination could be beneficial. A systematic review has suggested potential benefit of neoadjuvant chemotherapy, while other studies have demonstrated decreased overall survival and concluded that neoadjuvant chemotherapy prior to CRS for colorectal dissemination has no strong evidence for its efficacy regarding overall survival.^23^

In the metachronous peritoneal dissemination group, there was no significant difference in the prognosis between those who underwent CRS within 6 months and after 6 months. Preoperative chemotherapy was administered to 42 of 51 (82%) patients who underwent CRS within 6 months and 61 of 64 (95%) patients who underwent CRS after 6 months. Disease progression may be slower in patients with metachronous peritoneal dissemination owing to significantly more histologically differentiated types.

A systematic review showed that adjuvant chemotherapy, but not preoperative chemotherapy, contributes to improved survival rates after CRS.^23^ The present study also showed significantly improved survival in patients with either synchronous or metachronous dissemination who received adjuvant chemotherapy. However, in this study, a large number of patients received postoperative follow‐up at other hospitals instead of our institution, resulting in a significant number of patients with an unknown status regarding whether they received adjuvant chemotherapy after CRS. This may have had a substantial impact on the analysis of patient prognosis. Furthermore, the regimens and number of chemotherapy cycles varied. Multicenter randomized controlled trials to investigate the role of adjuvant chemotherapy are needed.

CRS with HIPEC is widely considered standard treatments for peritoneal dissemination of colorectal cancer. However, the effects of HIPEC on colorectal peritoneal dissemination remain controversial. One randomized study reported no significant difference in overall survival between CRS alone and CRS with HIPEC using oxaliplatin.^24^ We used mitomycin C as the chemotherapeutic drug for HIPEC in this study. Mitomycin C has been extensively used in intraperitoneal cancer chemotherapy for colorectal peritoneal dissemination, and its pharmacological actions and side effects have been thoroughly investigated. And mitomycin C is the only drug approved for intraperitoneal administration in Japan. We found that there was no significant difference in survival whether HIPEC was administered or not in cases of synchronous and metachronous dissemination. As a result of examining the factors associated with survival rates based on the presence or absence of HIPEC, only the preoperative tumor markers and time to CRS <6 months in patients who underwent CRS with HIPEC in the synchronous peritoneal dissemination group differed from the other factors. Because of the limited number of cases and variations in the observation periods, a larger number of cases and a longer observation period would be necessary to determine the actual involvement of HIPEC in survival rates and its associated factors. Furthermore, regarding HIPEC, several problems need to be solved such as the use of chemotherapeutic agents, time, and temperature. Several trials are currently being conducted regarding the usefulness of HIPEC, and their results are awaited.^25^

## 5 CONCLUSION

Although this was a single‐center retrospective study and the number of cases was relatively small, CRS for colorectal peritoneal dissemination is a useful surgical procedure for favorable long‐term prognoses for both synchronous and metachronous peritoneal dissemination. Case selection is extremely important to achieve satisfactory results, and the P classification and PCI can be useful indicators for both synchronous and metachronous cases. CRS should be considered in P1 and P2 cases or a PCI < 20, and detailed quantification of peritoneal dissemination by PCI is extremely important in P3 cases.

## FUNDING INFORMATION

None.

## CONFLICT OF INTEREST STATEMENT

The authors declare no conflicts of interest for this article.

## ETHICS STATEMENTS

Approval of the research protocol: N/A.

Informed Consent: N/A.

Registry and the Registration No. of the study/Trial: N/A.

Animal studies: N/A.
